# Development and characterization of silicone-based testosterone propionate implants for sustained androgen delivery in juvenile castrated male pigs

**DOI:** 10.1016/j.mex.2026.104009

**Published:** 2026-06-20

**Authors:** Sydney T. Puda, Heather A. Moeser, Adam J. Moeser

**Affiliations:** Department of Large Animal Clinical Sciences, College of Veterinary Medicine, Michigan State University, East Lansing, MI, USA

**Keywords:** Swine, Testosterone, Silicone implant

## Abstract

Validated techniques for androgen replacement in swine research models are limited, particularly for studies requiring controlled hormone delivery in males during early development. We adapted a silicone-based androgen implant methodology originally developed for adult female pigs to establish an androgen replacement model in castrated juvenile male pigs.

• Castrated male pigs were surgically implanted with low (0.5 g), medium (1.0 g), or high (2.0 g) doses of testosterone propionate (TP), and compared with castrated controls.
• Intact male pigs were included as a physiologically relevant reference group to benchmark implant-derived testosterone concentrations against endogenous androgen production during juvenile development.
• Circulating testosterone was monitored weekly for 49 days to evaluate the concentration and duration of testosterone release.
• Body weights were recorded weekly to assess body weight gain.

Testosterone propionate implants produced a dose-dependent elevation in circulating testosterone that peaked between 7 and ​14 days post-implantation and reached or exceeded concentrations of intact males. Following peak release, testosterone levels gradually declined across all implant groups but remained elevated relative to castrated controls throughout the 49-day study period, with the medium- and high-dose groups maintaining the highest concentrations. Growth rates of animals were similar across experimental groups, indicating that TP implantation did not adversely affect overall growth during the study. These findings validate silicone-based TP surgical implants as a reliable method for sustained androgen delivery in juvenile, prepubertal pigs, establishing a tractable model for mechanistic studies of androgen action with translational relevance to human and animal health.

## Graphical abstract

**Figure:**
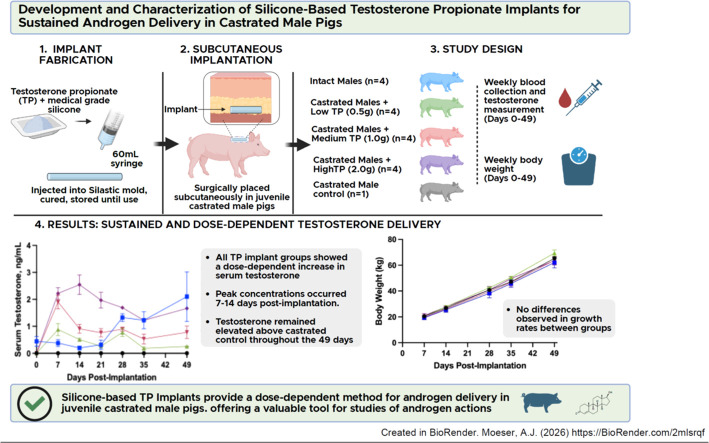

## Specifications table

**Table:** 

Subject area	Agricultural and Biological Sciences
**More specific subject area**	Endocrinology and Physiology
**Name of your method**	Silicone-based testosterone propionate (TP) implants for use in swine
**Name and reference of original method**	**Name:** Fabrication of testosterone enanthate silastic implants for use in large animals models
	**Reference:** Mahabir, N., & Newell-Fugate, A. E. (2024). Fabrication of silastic testosterone enanthate implants to achieve virilizing levels of serum testosterone in swine. *MethodsX, 12*, 102,549. DOI: 10.1016/j.mex.2024.102549
	https://doi.org/10.1016/j.mex.2024.102549
**Resource availability**	17β-(1-oxopropoxy)-androst-4-en-3-one (Trivial name: testosterone propionate; Sigma-Aldrich Cat # T1875–100 G)
	Analytical balance (Mettler Toledo Cat # ME54TE)
	Medical grade silicone adhesive (Factor II Cat # A-100)
	Weigh boat
	60 mL catheter tip syringe
	Dissecting scissors
	Nalgene Pharma-Grade Silicone Tubing, ID 0.250″, OD 0.375″ (Thermo Fischer Scientific Cat # 8600–0060)

## Background

Androgens play a critical role in male sexual differentiation and development. In males, serum androgen levels rise during distinct developmental periods which occur prenatally, postnatally, and at puberty. The prenatal and postnatal surges together comprise the perinatal androgen surge, a hormonally dynamic window essential for organizing male anatomy and physiology. Studies in rodents have shown that this surge not only directs sexual development, but also influences behavior [[1], [2], [3], [4], [5], [6], [7]], growth [[8], [9], [10]], and development of the immune systems-12 despite lasting <48 h [3,[11], [12], [13], [14], [15]]^,^. However, in humans, the perinatal androgen surge occurs within the first six months of life, peaking between one and three months [3,[16], [17], [18]].

Unlike rodents, swine exhibit key physiological and developmental similarities to humans. Notably, pigs experience a perinatal androgen surge that more closely resembles the human, lasting approximately three months and peaking within the first three to four weeks of lifetimes [[19], [20], [21]]. These developmental parallels highlight the potential of pigs as a translational model for studying androgen-mediated processes. In commercial swine production, male piglets are routinely castrated within the first ten days of life, eliminating gonadal androgen production during the perinatal period. Despite these advantages, swine remain underutilized in developmental endocrinology research, and validated methods for sustained hormone delivery are limited. Addressing this gap is essential for enabling mechanistic investigation of androgen-dependent programming in early life, and for establishing swine as a validated translational model in developmental endocrinology.

Mechanistic studies of androgen function demand precise control over hormone timing and dosage, requirements that are difficult to meet with current delivery approaches. In swine, testosterone replacement is typically administered via intramuscular injection [[22], [23], [24], [25]], requiring repeat injections every two to three days. This method produces frequent fluctuations in serum testosterone, generating peaks and troughs that diverge from the natural surge’s gradual rise and fall. These inconsistencies introduce confounding variables that can obscure mechanistic insight. Previous studies have employed testosterone or testosterone propionate implants in swine to address diverse biological and production-oriented questions [[26], [27], [28], [29]]. However, key parameters such as sustained hormone delivery, dose–exposure relationships, and longitudinal validation of circulating testosterone levels were not systematically assessed. This leaves important methodological questions regarding optimal formulation and dosing strategies for juvenile swine unresolved.

To address this gap, we tested whether a silicone-based testosterone propionate (TP) implant could produce sustained, physiologically relevant elevations in serum testosterone. We selected TP due to its relatively short half-life (∼1.5 days in human males [[30], [31], [32]]), which supports sustained yet non-accumulative delivery. This pharmacokinetic profile aligns with our study objective as hormone accumulation could obscure discernible peak-trough dynamics. Our implant fabrication methods were adapted from Mahabir and Newell-Fugate (2024), who used a similar silicone-based approach to deliver testosterone enanthate (TE) in female pigs [26]. Building on this foundation, we developed implants containing three different doses of TP and conducted a pilot evaluation to determine their feasibility for sustained androgen delivery in castrated male pigs. Here, we describe the development and pilot evaluation ofthis silicone-based testosterone propionate implant.

## Method details

### Animals and housing conditions

A total of 14 crossbred pigs (*Sus scrofa domesticus;* Yorkshire x Duroc) were used in this study. All animals were sourced from the Michigan State University Swine Teaching and Research Center. Male pigs were surgically castrated at 7 days of age, weaned at 21 days, and subsequently transported to an environmentally controlled nursery unit approximately one mile from the sow farm. Pigs were housed in 183 × 114-cm pens with tenderfoot flooring, accommodating three to four pigs per pen. Animals were housed with pen mates from the same treatment group to maintain consistency in post-operative management and minimize treatment-related behavioral confounding. Each pen was equipped with a single feeder, adjustable water nipples, and heat mats to provide supplemental warmth. Animals had ad libitum access to water and were fed a corn-soybean meal-based diet formulated to meet or exceed NRC nutrient requirements for each developmental stage. Environmental conditions were carefully maintained to support thermoneutrality, with nursery conditions held at approximately 29 °C and grower conditions at 24 °C. Room temperature and humidity were recorded daily, and lighting was maintained on a 12:12-hour light-dark cycle. Animal health and welfare was monitored daily, with assessments including physical appearance, activity level, alertness, body condition, and behavior.

### Experimental design and treatment groups

The study included both castrated and intact pigs to evaluate the ability of TP implants to elevate serum testosterone to physiologically relevant levels. Castrated pigs were assigned to one of four treatment groups: low dose (0.5 g TP; n = 2), medium dose (1.0 g TP; n = 3), high dose (2.0 g TP; n = 2), and a negative control group (n = 1) that received no hormone implant. To provide a reference for natural androgen levels, intact male pigs (n = 4) were included as physiological controls.

### Implant fabrication

Testosterone propionate (Sigma-Aldrich, St. Louis, MO, Cat # T1875–​100 G) was incorporated into a medical-grade silicone matrix using Medical Silicone Adhesive (Factor II, Incorporated, Lakeside, AZ, Cat # A-100). Silastic tubing (Nalgene Pharma-Grade Silicone Tubing, ID 0.250″, OD 0.375″; Thermo Fischer Scientific, Waltham, MA, Cat # 8600–0060) was used as the mold for implant formation. All materials were handled and weighed using a Mettler Toledo analytical balance (Model MS104S, readability: 0.0001 g; Columbus, OH, USA, Cat # ME54TE).

Given the relatively smaller size of the pigs used in this study compared with Mahabir and Newell-Fugate (2024) ^26^, we initially attempted to formulate a 50:50 TP to silicone mixture by weight to decrease the length of the implant but resulted in a paste with excessive viscosity unsuitable for loading into tubing. Therefore, we adopted the 20:80 TP to silicone ratio by weight following a previous method [26]. Through pilot testing, we determined that mixing 1 g of TP with 5 g of silicone yielded a 10 cm implant when loaded into the tubing mold. These 10 cm implants were subsequently cut in half to create standardized 5 cm implants, each containing 0.5 g TP, with medium and high dose animals receiving multiple 5 cm implants.

To prepare the implants, the required amount of silicone adhesive was weighed in a disposable weigh boat, followed by the addition of the appropriate amount of TP. The two components were mixed thoroughly by hand using a spatula until uniform consistency was achieved. The mixture was then transferred into a 60 mL plastic catheter tipped syringe, which had its tip trimmed to allow a snug fit into the silastic tubing. Tubing segments were pre-cut to 13 cm lengths to facilitate handling and loading. The syringe was inserted into the tubing and used to fill each segment with the TP-silicone mixture to a length of approximately 10 cm. The filled tubes were then stored at room temperature to allow the silicone to cure.

After 48 h, implants were carefully removed using blunt dissection and scissors. Implants were sterilized by immersion in a chlorhexidine solution for 12 h and subsequently transferred to 70% ethanol for 1–2 h immediately prior to implantation.

### Surgical implant

At 43 days of age, silicone-based implants containing testosterone propionate (TP) were placed subcutaneously in the dorsal cervical region (between the shoulder blades). This anatomical site was selected to minimize interference with normal activity and to ensure consistent implant placement across animals. Pigs were anesthetized with an intramuscular injection of TKX, a combination of Telazol® (tiletamine + zolazepam), Ketamine, and Xylazine. TKX was administered at a dose of 0.4 mL/kg, corresponding to approximately (0.8 mg/kg) of Telazol, (3.7 mg/kg) of Ketamine, and (0.5 mg/kg) of Xylazine. Blood samples were collected via jugular venipuncture before surgical preparation, and adequate anesthetic depth was confirmed by lack of response to surgical incision.

The surgical site was shaved and antiseptically prepared using alternative scrubs of dilute chlorhexidine and 70% ethanol. A midline skin incision approximately 3–4 cm in length was made with a 10-blade between the shoulder blades, followed by blunt dissection caudally and laterally to create a subcutaneous pocket for implant placement. Implants were inserted subcutaneously based on the assigned dosage group: low dose pigs (0.5 g TP) received one implant placed on the left side, medium dose pigs (1.0 g TP) received one implant placed on the left side and one on the right side, and high dose pigs (2.0 g TP) received two implants placed on the left side and two on the right side.

The subcutaneous and skin layers were closed using 3–4 simple interrupted sutures with 2/0 PDS to ensure proper tissue approximation. All implanted animals received an injection 0.7 mL Meloxicam IM for pain management while they were anesthetized. Following surgery, pigs were placed in individual recovery pens and monitored until fully alert, standing, and ambulatory. Once stable, pigs were returned to their home pens with pen mates. Post-operative monitoring was performed twice daily for the duration of the study. Observations included behavioral assessments (appetite, water intake, mobility), overall health, and examination of the implant site for signs of infection or other complications. Sutures were removed 14 days post-surgery, following confirmation of complete incision healing.

### Sampling procedures

Blood samples and body weights were collected weekly throughout the study. At 49 days post-implantation, pigs were humanely euthanized and terminal serum samples along with final body weights were obtained. For blood collection, pigs under 35 kg were restrained in dorsal recumbency and blood was drawn from the jugular vein using 20 G x 1 ¼” Vacuette® needles into red-top serum tubes. Pigs exceeding 35 kg were restrained using a snare to ensure safe and effective sampling. Immediately following blood collection, pigs were guided from their pens and weighed using an Arlyn Series 320D-27 industrial veterinary scale (Arlyn Scales, Rockaway, NY, USA; readability: 0.01 kg).

Collected blood samples were allowed to sit at room temperature for 30 min, then centrifuged at 2700 RPM at 8 °C for 30 min. Serum was carefully separated into 1 mL aliquots, initially stored at −20 °C for 48 h and subsequently transferred to a − 80 °C freezer for long-term storage. Aliquots were shipped to BET laboratories (Lexington, KY) for testosterone quantification via radioimmunoassay (RIA).

### Data analysis

**Fig. 1:**
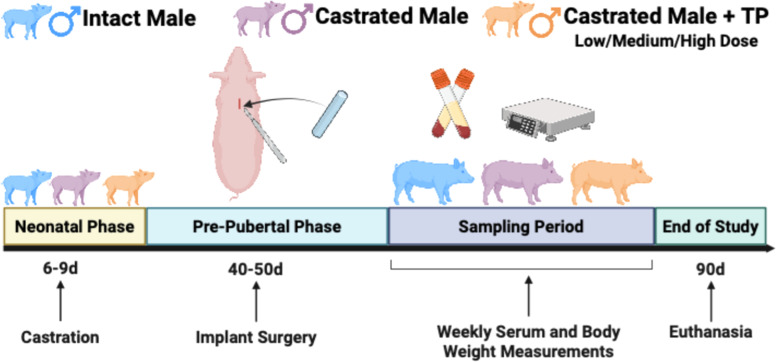
Experimental timeline for pigs. Male piglets were castrated at 6–9 days of age or left intact and were weaned from their dam at 21 days. Testosterone implants were administered to some castrated males between 40–50 days of age, and pigs were maintained for 49 days. Blood was collected weekly throughout the trial to monitor serum testosterone concentrations.

**Fig. 2:**
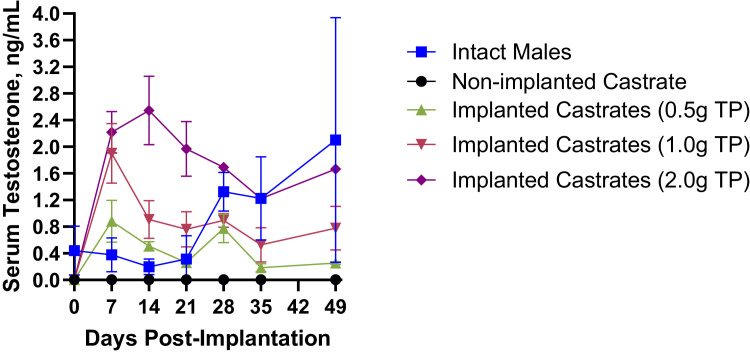
Serum testosterone levels. Mean (± SD) serum testosterone concentrations in intact males (n = 4), non-implanted castrate (n = 1), and implanted male castrates implanted with 0.5 g TP (n = 2), 1.0 g TP (n = 3), and 2.0 g TP (n = 2).

**Fig. 3:**
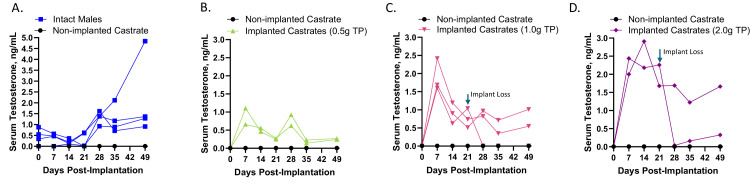
Individual serum testosterone levels. Weekly serum testosterone concentrations over 49 days in intact males, a non-implanted castrate, and TP-implanted castrates. Each line represents an individual animal. (A) Intact males vs non-implanted castrate. (B) 0.5 g TP–implanted castrates. (C) 1.0 g TP–implanted castrates. (D) 2.0 g TP–implanted castrates. Arrows denote implant loss events.

Due to the exploratory and pilot nature of this study and the limited number of animals per treatment group (n = 2–3), statistical analyses were not performed. Instead, circulating testosterone concentrations and body weights are presented as group means ± standard error of the mean (SEM) to illustrate overall trends, dose responsiveness, and temporal patterns of hormone release. During the study, two pigs experienced implant expulsion events, which resulted in abrupt declines in circulating testosterone concentrations. Because serum testosterone values obtained after confirmed implant loss no longer reflected active hormone delivery, these post-expulsion timepoints were excluded from group mean calculations (Fig. 1, Fig. 2) intended to characterize implant performance. Data collected prior to implant expulsion were retained. Post-expulsion testosterone values are shown in individual animal plots to illustrate the dependence of circulating hormone levels on implant retention (Fig. 3).

## Method validation

### In *vivo*validation of testosterone implants

All three implant doses produced sustained, dose-dependent elevations in serum testosterone concentrations, whereas testosterone remained undetectable in the castrated control pig throughout the study (Fig. 2, Fig. 3). Following implantation, TP implants produced a rapid rise in circulating testosterone, with concentrations increasing within the first week and peaking between days 7 and 14 in a dose-dependent manner (Fig. 2, Fig. 3). Peak serum testosterone concentrations were highest in the high-dose (2.0 g) group, intermediate in the medium-dose (1.0 g) group, and lowest in the low-dose (0.5 g) group. After reaching peak levels, circulating testosterone concentrations declined gradually across all implant doses. Despite this decline, low-, medium- and high-dose implants maintained elevated testosterone concentrations relative to their baseline and the non-implanted castrate. Individual animal trajectories confirmed consistent implant performance and reproducible testosterone delivery across animals (Fig. 3). Across doses, all implanted piglets achieved serum testosterone concentrations comparable to or exceeding intact males during the first 3–4 weeks post-implantation. Implant expulsion events resulted in immediate and marked reductions in circulating testosterone, directly linking hormone levels to implant retention. One medium-dose pig expelled both implants and one high-dose pig partially expelled implants at approximately day 28. These animals were retained in the individual pig data plots (Fig. 3), where implant expulsion accounted for the abrupt declines observed at later time points. Despite inter-animal variability, serum testosterone concentrations followed a consistent dose-dependent trajectory across treatment groups. Intact males displayed lower androgen concentrations at study onset, consistent with the post-neonatal period, and one intact male exhibited an increase between study days 40–49, reflecting the initial onset of peri‑pubertal androgen production. Importantly, all implant doses achieved serum testosterone concentrations equal to or greater than those of intact males through day 21 and remained elevated relative to baseline and the castrated control animal, demonstrating continual androgen delivery over the 7-week experimental period. Taken together, this silicone-based TP implant method offers several advantages and broad applications. It provides a reproducible and dose-controllable approach for sustained androgen delivery in juvenile male pigs. The implant reduces the need for repeated injections, and testing multiple implant doses provides insight into dose-response relationships and optimal hormone delivery strategies. Among the doses tested, the medium-dose (1.0 g TP) implant appeared to provide the most practical balance between sustained testosterone elevation, implant burden, and maintenance of circulating hormone concentrations within a physiologically relevant range. Although the high-dose implants produced the greatest testosterone exposure, they required placement of multiple implants and may have exceeded physiologic concentrations during peak release. Beyond mechanistic studies of androgen action, this model has applicability for investigating developmental programming, metabolic processes, behavior, and translational studies relevant to both human and animal endocrine health and disease.

### Growth performance and health

**Fig. 4:**
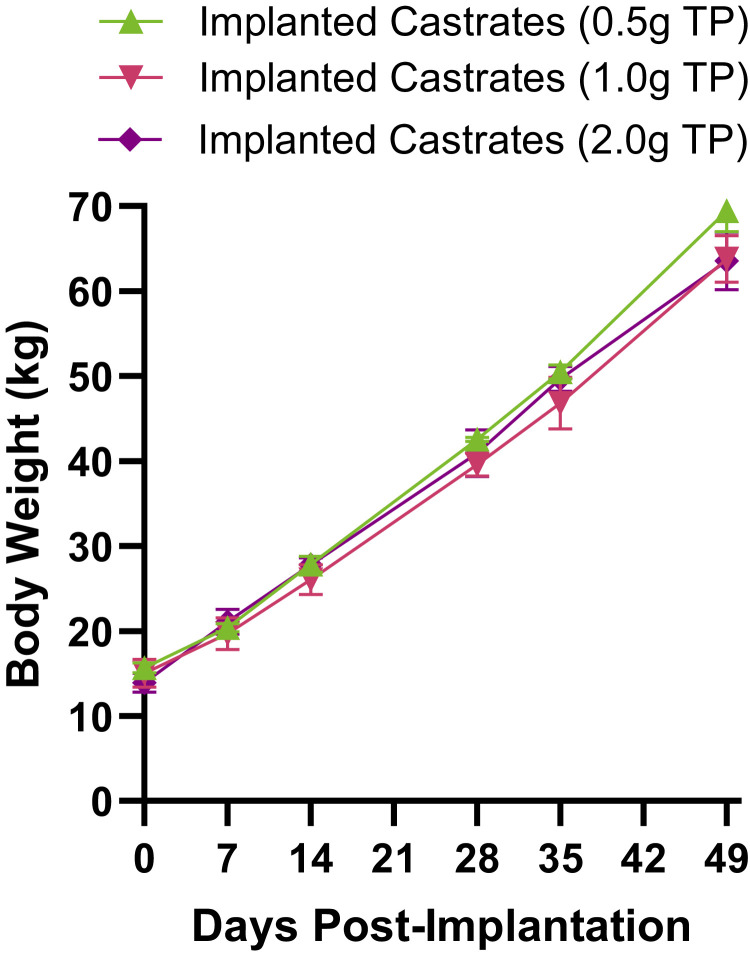
Body weight gain for implanted pigs. A: Weekly body weights (mean ± SD) measured over 49 days in TP-implanted castrates (0.5 g, 1.0 g, 2.0 g).

All animals in the study gained weight over the 49-day period (Fig. 4). Body weights and growth trajectories were within the expected range for pigs of this age and breed, indicating normal health and development across all groups. The overall growth rates were not affected by treatment (Table 1). Although testosterone supplementation did not produce detectable differences in body weight gain during the study period, the relatively young age of the animals, short experimental duration, and prepubertal developmental stage may have limited the manifestation of anabolic or growth-related effects typically associated with androgen exposure. In addition, no overt aggression or abnormal social behaviors were observed. However, the males in this study were prepubertal and thus many secondary behavioral and social characteristics associated with testosterone exposure may not yet be fully expressed or organized at this developmental stage. Longer-term studies extending into later developmental stages may be necessary to evaluate the full physiological and behavioral consequences of sustained androgen supplementation in swine.

**Table 1:** Methods validation.

Group	n	Total BW gain (kg)	ADG (g/day)
Non-implanted castrate	1	44.92	1070
Intact males	4	42.71 ± 2.77	1017 ± 66
Implanted castrates (0.5 g TP)	4	46.39 ± 3.64	1105 ± 87
Implanted castrates (1.0 g TP)	3	43.22 ± 1.89	1029 ± 45
Implanted castrates (2.0 g TP)	2	42.48 ± 1.97	1011 ± 47

Table Legend: Total BW gain and Average Daily Gain (ADG) (Day 7–49) presented as mean ± SD for all experimental groups.

## Limitations

Several limitations that warrant consideration emerged throughout this study. First, implant formulation required a 20:80 TP to silicone ratio, resulting in relatively long implants. For studies involving very young or small piglets, the number and size of implants needed may pose practical challenges. Second, this method is invasive, requiring surgical placement under anesthesia and appropriate veterinary oversight. Due to the limited subcutaneous space in juvenile pigs, placement of multiple implants was challenging. Post-surgical complications, including implant-site infection, dehiscence, and partial implant loss occurred. One piglet was euthanized due to a persistent implant-site infection that failed to resolve despite continued growth and otherwise normal health. The infection did not resolve with medical intervention, including drain placement and systemic antibiotics. Future optimization of this method may include development of shorter or lower-volume implant formulations, alternative silicone-to-TP ratios, or modified implant materials designed to improve implant retention and reduce post-surgical complications.

## Ethics statements

All animal procedures were conducted in strict accordance with institutional guidelines for the care and use of laboratory animals and were approved by the Michigan State University Institutional Animal Care and Use Committee (IACUC Protocol #PROTO202200464). The study adhered to the ARRIVE guidelines and was carried out in accordance with the National Institutes of Health Guide for the Care and Use of Laboratory Animals (NIH Publication No 8023, revised 1978). Animal welfare was prioritized throughout the study, including all aspects of housing, handling, and experimental intervention. The sex of all animals was male, and castration status was used as an experimental variable.

## Related research article

None

## CRediT authorship contribution statement

**Sydney T. Puda:** Formal analysis, Investigation, Methodology, Validation, Visualization, Writing – original draft. **Heather A. Moeser:** Conceptualization, Methodology, Writing – review & editing. **Adam J. Moeser:** Conceptualization, Funding acquisition, Investigation, Methodology, Project administration, Resources, Supervision, Validation, Visualization, Writing – original draft, Writing – review & editing.

## Declaration of competing interest

The authors declare that they have no known competing financial interests or personal relationships that could have appeared to influence the work reported in this paper.

## Data Availability

Data will be made available on request.
